# First clinical study of a pegylated diabody ^124^I-labeled PEG-AVP0458 in patients with tumor-associated glycoprotein 72 positive cancers

**DOI:** 10.7150/thno.49422

**Published:** 2020-09-15

**Authors:** Andrew M. Scott, Timothy Akhurst, Fook-Thean Lee, Marika Ciprotti, Ian D. Davis, Andrew J. Weickhardt, Hui K. Gan, Rodney J. Hicks, Sze Ting Lee, Pece Kocovski, Nancy Guo, Maggie Oh, Linda Mileshkin, Scott Williams, Declan Murphy, Kunthi Pathmaraj, Graeme J. O'Keefe, Sylvia J. Gong, John S. Pedersen, Fiona E. Scott, Michael P. Wheatcroft, Peter J. Hudson

**Affiliations:** 1 Olivia Newton-John Cancer Research Institute and School of Cancer Medicine, La Trobe University, Heidelberg, Victoria 3084, Australia.; 2 Department of Molecular Imaging and Therapy, Austin Hospital, and University of Melbourne, Heidelberg, Victoria 3084, Australia.; 3 Ludwig Institute for Cancer Research, Melbourne Branch, Heidelberg, Victoria 3084, Australia.; 4 Centre for Cancer Imaging, the Peter MacCallum Cancer Centre, Melbourne, Victoria 3000, Australia.; 5 The Sir Peter MacCallum Department of Oncology, the University of Melbourne Melbourne, Victoria 3000, Australia.; 6 Eastern Health, Melbourne, Australia.; 7 Monash University, Melbourne, Australia.; 8 Department of Medical Oncology, Austin Health, Heidelberg, Victoria 3084, Australia.; 9 Avipep Pty Ltd and the Victorian Cancer Biologics Consortium, Parkville, Victoria 3052, Australia.; 10 TissuPath Specialist Pathology, Mount Waverley, Victoria 3149, Australia.

**Keywords:** pegylated diabody, TAG-72, first-in-human, biodistribution, PET imaging

## Abstract

Through protein engineering and a novel pegylation strategy, a diabody specific to tumor-associated glycoprotein 72 (TAG-72) (PEG-AVP0458) has been created to optimize pharmacokinetics and bioavailability to tumor. We report the preclinical and clinical translation of PEG-AVP0458 to a first-in-human clinical trial of a diabody.

**Methods:** Clinical translation followed characterization of PEG-AVP0458 drug product and preclinical biodistribution and imaging assessments of Iodine-124 trace labeled PEG-AVP0458 (^124^I-PEG-AVP0458). The primary study objective of the first-in-human study was the safety of a single protein dose of 1.0 or 10 mg/m^2 124^I-PEG-AVP0458 in patients with TAG-72 positive relapsed/ metastatic prostate or ovarian cancer. Secondary study objectives were evaluation of the biodistribution, tumor uptake, pharmacokinetics and immunogenicity. Patients were infused with a single-dose of ^124^I labeled PEG-AVP0458 (3-5 mCi (111-185 MBq) for positron emission tomography (PET) imaging, performed sequentially over a one-week period. Safety, pharmacokinetics, biodistribution, and immunogenicity were assessed up to 28 days after infusion.

**Results:** PEG-AVP0458 was radiolabeled with ^124^I and shown to retain high TAG-72 affinity and excellent targeting of TAG-72 positive xenografts by biodistribution analysis and PET imaging. In the first-in-human trial, no adverse events or toxicity attributable to ^124^I-PEG-AVP0458 were observed. Imaging was evaluable in 5 patients, with rapid and highly specific targeting of tumor and minimal normal organ uptake, leading to high tumor:blood ratios. Serum concentration values of ^124^I-PEG-AVP0458 showed consistent values between patients, and there was no significant difference in T½α and T½β between dose levels with mean (± SD) results of T½α = 5.10 ± 4.58 hours, T½β = 46.19 ± 13.06 hours.

**Conclusions:** These data demonstrates the safety and feasibility of using pegylated diabodies for selective tumor imaging and potential delivery of therapeutic payloads in cancer patients.

## Introduction

Numerous antibody-based cancer therapies have been developed, taking advantage of the specificity of targeting tumor antigens and advances in recombinant protein production technology. Antibody therapeutics can provide significant benefit to some patients with cancer, either as monotherapies, or in combination with drugs and radiotherapy 1-3. Antibodies have also been conjugated to drugs and radionuclides to provide targeted delivery of these payloads, enabling superior efficacy and/or reduced payload toxicity 4-6. This approach to cancer therapy has been facilitated by advances in radiochemistry, linker, and payload technology 2,4. Radioimmunoconjugates have been shown to be useful for both detection of tumors, and for therapy 6, 7. The long half-life of intact antibodies limits the dose to tumor due to enhanced red marrow toxicity, leading to exploration of new strategies to improve the therapeutic index 7. Radionuclide targeted therapy of tumors has also been supported by the recent success of peptide-based strategies, including ^177^Lu-DOTATATE in neuroendocrine tumor patients, and ^177^Lu-PSMA-617 in prostate cancer patients 8-9. Radioimmunoimaging of tumor sites may also provide additional information on the stage of disease, *in-vivo* evidence of expression of antigen by tumor, and subsequent likely response to targeted monoclonal antibody-based therapeutic approaches through a theranostics approach 6,10-13.

Multimeric antibody fragments (e.g. diabodies, triabodies, minibodies) represent an alternative to intact antibodies as they are characterized by increased *in vivo* tissue penetration, high avidity (slow off-rates) and faster blood clearance 7, 14,15. These properties make them more attractive for imaging with shorter-lived radioisotopes suited for positron emission tomography, as well as for payload delivery. For the diabody format, scFv molecules with short (4-5 amino acid) linkers between their variable heavy (V_H_) and variable light (V_L_) chains form stable noncovalent dimers of approximately 55kDa in size 14-17. Diabodies, like intact antibodies, retain two antigen binding regions, which enables them to attain very high avidity for the target antigen. In animal models, these bivalent diabodies exhibited high tumor uptake, but substantial kidney uptake due to passive clearance and retention, and rapid blood clearance 7,18-20.

One approach to improve the bioavailability of multimeric antibody fragments is through pegylation of surface lysine residues to increase the apparent molecular size of diabodies and avoid first-pass renal clearance, thus extending the half-life in circulation and theoretically increasing tumor uptake 21-23. The AVP04 diabody used in this study is derived from the murine monoclonal antibody CC49, which has been evaluated in clinical trials targeting the tumor associated glycoprotein 72 antigen (TAG-72) 24-28. TAG-72 is a glycoprotein expressed on the surface membrane of many cancer types, including colon, ovarian, lung, breast and prostate cancers, but is not expressed in normal tissues apart from secretory endometrium, and fetal tissues 24-29. Initially, using random surface conjugation of PEG to lysine residues, the radiolabeled AVP04 diabody generated promising xenograft uptake data, although the lysine pegylation produced a heterogeneous and uncontrolled product population and potentially impair binding affinity 30. Using molecular modelling and surface accessibility calculations two cysteine residues were introduced to generate a unique surface disulphide at positions 8-11 of the V_L_-domain, and pegylation was then specifically directed to surface these cysteine residues 31. However, PEG conjugation utilizing vinyl sulphone chemistry was incomplete and resulted in less than the 4 expected PEG adducts 31. This His6-tag specifically-pegylated diabody product achieved a significant improvement in xenograft tumor uptake up to 70%ID/g (percent injected dose per gram) compared to 50%ID/g for diabodies with random lysine pegylation 31.

We now report an improved conjugation strategy, using maleimide chemistry, to achieve stoichiometric pegylation of exactly four PEG24 molecules per diabody (PEG-AVP0458), which increased the molecular weight from 52kDa to 56kDa and the molecule's hydrodynamic radius. PEG24 was chosen based on our prior results which showed that PEG12 had slightly higher kidney uptake than PEG24 and PEG48 in a mouse model, and we selected PEG24 for surety in reducing kidney clearance in humans 31. This precisely pegylated diabody was then analyzed in preclinical biodistribution studies using TAG-72 positive human cancer xenografts in mice and molecular PET imaging using ^124^I-PEG-AVP0458. We have then explored this improved pegylated diabody in a first-in-human clinical biodistribution trial, and demonstrate ^124^I-PEG-AVP0458 to be safe, with high, specific targeting of TAG-72 expressing tumors in prostate cancer patients. This clinical trial is the first assessment in man of a monospecific, bivalent diabody, specifically designed for cancer theranostics. These data support the the development of PEG-AVP0458 (or PEG-avibody constructs) as a payload delivery platform, and for theranostic use in patients.

## Methods

### Production and characterization of PEG-AVP0458

AVP0458 is a recombinant scFv fragment derived from the parent CC49 antibody 24,32 and comprises 234 amino acid residues in VH-linker-VL orientation in which the short GGGGS linker prevents Fv folding and instead directs dimerization to form a 'diabody' with two antigen-binding Fv arms. Within the framework (non-binding) regions of the antibody, residues 8 and 11 in VL were substituted with cysteine residues to provide two stable surface-exposed disulphides 31. The pairs of residues were carefully selected from structural modelling to promote the formation of disulphide binds between then so as to avoid the presence of free thiols that might lead to protein aggregation. The recombinant AVP0458 protein was produced in three 10L batches under cGMP conditions using intracellular expression in Eschericia coli and recovered by mild disruption of inclusion bodies (Hospira Adelaide Ltd, Adelaide, Australia). Purification involved a protein refolding step followed by three, sequential chromatographic purifications. The three chromatography steps after refolding used cation-exchange chromatography on an SP-BB column, anion-exchange chromatography by passing the material through a QFF column to reduce endotoxin levels, and a final cation-exchange chromatography purification step on an SP-HP column. The two surface disulphides were then reduced and conjugated with four maleimide-PEG24 chemical moieties per diabody, using single molecular weight, linear, GMP grade dPEG^TM^ (Quanta Biodesign, Ohio, USA). This resulted in a final PEG-AVP0458 drug substance with molecular weight of 56,044 Da. After a final purification by cation exchange chromatography, PEG-AVP0458 was concentrated and buffer exchanged into phosphate buffered saline by tangential flow filtration.

Antigen binding activity of the PEG-AVP0458 diabody compared to the parental CC49 antibody was assessed by surface plasmon resonance binding to bovine submaxillary mucin (BSM) the standard antigen mimetic for the TAG-72 sialyl-Tn antigen, using cross competition analyses in a BIAcore 2000 (BIAcore AB, Uppsala, Sweden). Apparent binding constant *K*d rate constants were calculated by nonlinear least squares regression analysis, using BIAevaluation version 3.0 software. Comparison of CC49 and AVP0458 binding to tumor tissue was performed by immunohistochemical analyses on 10 serial (adjacent) sections of formalin-fixed, paraffin-embedded Gleason 3+4 prostate cancer tissue. The murine CC49 antibody tissue binding pattern was detected with an anti-murine IgG-HRP detection system and AVP0458-biotin binding determined with anti-biotin detection. A 14-day toxicity study of a single intravenous dose of PEG-AVP0458 in Sprague Dawley rats was also conducted (RDDT Laboratories, Bundoora, VIC, Australia) in compliance with GLP requirements.

### Synthesis and characterization of ^124^I-PEG-AVP0458

The radiolabeling of PEG-AVP0458 was performed by attaching ^124^I (Austin Health, Melbourne, Australia) to the tyrosine residues (Y) of diabody using the IODOGEN method (Pierce Inc., Rockford, IL, USA) under sterile conditions. Briefly, ^124^I (in 0.0225M NaOH) was neutralised by mixing with equal volume of 0.5M potassium phosphate buffer, pH 7.2. PEG AVP0458 and iodogen coated glass beads were added, and gently mixed while incubated at ambient temperature. The radiolabeled product was purified from the mixture using a disposable 15mL column containing Sephadex G50-80 (Sigma-Aldrich, Sydney, Australia) equilibrated in saline and assayed using a dose calibrator and radio thin layer chromatography. The radioimmunoreactivity was determined on TAG-72 expressing LS-174T carcinoma cells using Lindmo and Scatchard analyses as previously described 33, and specific activity, purity, radiochemical yield of the ^124^I-bound PEG-AVP0458 were determined for each preparation prior to infusion.

### Preclinical model studies

Preclinical biodistribution protocols were approved by the Austin Health Animal Ethics Committee and performed according to the Australian national guidelines and standards in BALB/c nude mice bearing TAG-72 expressing LS-174T human carcinoma xenografts. On Day 0 a single dose of ^124^I-PEG-AVP-0458 (5 µg/16 µCi (0.59 MBq) in 0.1 ml 0.05% w/v HSA in saline) was administered via the tail vein to mice bearing established xenografts. At designated time points (Days 0, 1, 2, 3 and 6) groups of mice (n=5) were euthanized then organs, blood and tumors were collected for biodistribution assessment by gamma counting of tissue as previously described 30. For PET imaging, a separate group of 5 tumor bearing mice were injected with 58 µCi (2.15 MBq) of the same radioconjugate. PET/CT images were recorded at Day 0 (2 hours post injection), Day 1, Day 3 and Day 6 using a Philips Allegretto small animal PET scanner and Gemini CT scanner (Philips Healthcare, Cleveland, OH, USA).

### Clinical trial design

A Phase I open label first-in-human study of the safety and biodistribution of two doses of PEG-AVP0458, labeled with 3-5 mCi (111-185 MBq) of ^124^I for PET imaging was conducted at 2 sites in accordance with the IRB approved clinical trial protocol (Australian and New Zealand Clinical Trials Registry No: ACTRN12612000802808) and the Declaration of Helsinki and the ICH Guidelines for Good Clinical Practice. All patients provided written informed consent. Primary objectives were to assess the safety of single dose ^124^I-labeled PEG-AVP0458 (^124^I-PEG-AVP0458) in patients with TAG-72 positive ovarian or prostate cancer confirmed by immunohistochemical analysis of archived tumor specimens. The secondary objectives of this study were to evaluate the biodistribution, including tumor targeting, pharmacokinetics (PK) and immunogenicity of ^124^I-PEG-AVP0458.

Patients with relapsed or refractory ovarian cancer, metastatic prostate cancer, or primary prostate cancer who were pre‑prostatectomy or pre-radiotherapy, were eligible for enrollment if they were ≥ 18 years old with histologically proven TAG-72 positive, measurable disease, expected survival ≥ 3 months, ECOG performance status 0-1, and able to give written informed consent. Positive TAG-72 expression was defined as >20% of cancer cells staining positive for TAG-72 by immunohistochemistry. Additional inclusion criteria included adequate bone marrow, liver and renal function. Patients on regular corticosteroid, nonsteroidal anti-inflammatory drug or other immunosuppressive treatment including radiation or biological therapies or other investigational products within 4 weeks prior to first drug administration, or likely to be required during the 4 week on study period, were excluded. Additional exclusion criteria for individuals who had medical illnesses unrelated to their cancer included pregnancy, lactation, unstable cardiac disease, active infections requiring antibiotics and bleeding disorders.

Following pretreatment assessments, eligible patients received a single infusion of ^124^I-PEG-AVP0458 at a protein dose level of 1.0 or 10 mg/m^2^ administered intravenously in 100 mL of normal saline containing 5% human serum albumin over 1 hour on day 0. Patients were monitored for safety (including vital signs) during and for 4 hours post infusion, and on each day of visit until end of study (Day 28). Safety of ^124^I-PEG-AVP0458 was determined by the monitoring of the incidence and intensity of adverse events graded using the National Cancer Institute Common Terminology Criteria for Adverse Events (CTCAE) v4.0.

^124^I was used to trace label PEG-AVP0458, allowing whole body PET/CT imaging to assess biodistribution and tumor targeting. To block free uptake of free iodine by the thyroid, super saturated potassium iodide (SSKI), 10 drops orally, was self-administered twice a day for 10 days, commencing prior to infusion of ^124^I-PEG-AVP0458. Blood samples were also evaluated in all patients for hematologic, renal and liver function at pre study screening, and on Day 14 and 28. Blood was also taken for pharmacokinetics of ^124^I-PEG-AVP0458 and ELISA analysis of sera PEG-AVP0458 protein levels at baseline and at each study visit to Day 14, and for ELISA also on Day 21 and 28. Blood samples for immune response (human anti-diabody antibody) were taken at baseline, and Days 6, 14, 21 and 28. PET imaging was performed post infusion of ^124^I-PEG‑AVP0458 on Day 0, and on further 4 occasions over a one-week period.

### PET/CT image acquisition

At both trial sites, a non-contrast low dose CT (ldCT) scan were obtained prior to the acquisition of each whole-body PET scan on a GE Discovery^TM^ PET/CT 690 imaging system (GE Healthcare, Chicago, IL, USA) or Phillips Gemini^TM^ TF PET/CT imaging system (Philips Healthcare, Cleveland, OH, USA). The PET scans were acquired on Day 0, Day 1 and Day 2/3, and Day 4/5 and Day 6/7. On the GE Discovery PET/CT 690, scans were acquired in histogram mode and except for the leg regions, scan durations for each bed position for days 0, 1, 2/3 and 4/5 were respectively, 3, 3, 4, and 5 mins. For the leg regions, the scan duration was reduced to 1-min. The Philips Gemini TF64 acquisitions were acquired in listmode and the scan durations for each bed position for days 0, 1, 2/3 and 4/5 were respectively 3, 3, 3, and 4 min. For both systems, the total scan duration was nominally 30 mins.

A 5 mL cylindrical source with 0.14 mCi (5.12MBq) of ^124^I-PEG-AVP0458 was placed opposite the right foot as an imaging standard and scanned with the patient on each time point to confirm the routine calibration of scanner sensitivity. Within each subject, the VOI (Volume of Interest) analyses of the standards were constant to within 1%. Across all subjects, the VOI analyses of the standards were constant to within 6%. The resultant calibration correction factor was 0.84 +/- 0.05.

The acquired PET data were reconstructed using the vendor provided reconstruction frameworks, each correcting for half-life decay, deadtime and attenuation effects, randoms and scatter contributions. For the GE Discovery PET/CT 690, an OSEM algorithm utilizing 2 iterations, 18 subsets and a post reconstruction gaussian 7 mm FWHM filtering was used. The resultant image data were reconstructed onto a 192x192 matrix with a pixel size of 3.385 mm and a slice thickness of 3.27 mm. The Philips Gemini TF utilised a listmode-RAMLA wholebody reconstruction protocol which consisted of 3 iterations and a relaxation setting of 0.7 with the remaining RAMLA parameters set to the manufacturer defaults. The resultant image data were reconstructed onto a 144×144 matrix with a pixel size and slice thickness of 4 mm.

### Image analysis

Qualitative analysis of PET/CT images were performed by two experienced Nuclear Medicine physicians. Parameters assessed included normal organ uptake and clearance, and tumor uptake of reference lesions (scored on a 0-3 point scale). The low dose CT images were used as the anatomical reference to define the Regions of Interest (ROIs) of the imaging standard, whole body, organ and tumor in each patient, for each imaging timepoint. ROIs were then analysed for uptake and residence time of ^124^I-PEG-AVP0458 in whole-body, organs and tumors following the infusion doses.

### Pharmacokinetics

#### ^124^I-PEG-AVP0458

Serum obtained from patients following infusion of ^124^I-PEG-AVP0458 was aliquoted and radioactivity was measured with an automated gamma counter (Wizard, PerkinElmer, Australia). The results were expressed as % injected dose per liter (%ID/L) and μg/mL. A 2 compartment IV bolus model with macro-parameters, no lag time and first order elimination (WNL Model 8) was fitted to individual labelled infusions for each subject using un-weighted non-linear, least squares with Phoenix WinNonLin (Certara, St Louis, MO, USA).

#### PEG-AVP0458

A validated sandwich enzyme-linked-immunosorbent assay (ELISA) method was used to measure PEG-AVP0458 protein concentrations in sera. Briefly, the method entailed immobilization of the TAG72 antigen on microplates, blocking non-specific binding sites on the microtiter with SuperBlock^TM^ blocking buffer in PBS (Thermo Fisher Scientific, Waltham, MA USA), then capture of the PEG-AVP0458 diabody from solution. The 'captured' diabody was then targeted using a commercially available rabbit anti-PEG monoclonal antibody which reacts with the PEG component of the AVP0458-PEG molecule. The anti-PEG monoclonal antibody was in turn detected using a Goat Anti Rabbit IgG (H+L) Horseradish Peroxidase conjugate secondary antibody and ABTS as a colourimetric substrate. Pharmacokinetic parameters were determined using PK Solutions software (Version 2.0.7 for Mac, Summit Research Services, Montrose, CO, USA).

### Immunogenicity

Human Anti-Diabody AVP-0458 antibodies (HADAs) were measured by with a validated ELISA protocol. Briefly, the method involved the coating of ELISA microplates with the PEG-AVP0458 or human IGF-II proteins as the capture antigens. The non-specific binding sites on the microplate were then blocked. The PEG-AVP0458 containing-wells were then incubated with the test human serum samples and the IGF-II wells with a human IGF-II antibody. Subsequently a secondary anti-human antibody horseradish peroxidase conjugate was used to detect the presence of human antibodies using a standard TMB substrate colorimetric method. A positive signal in the IGF-II containing wells is used in the absence to human anti-PEG-AVP0458 antibodies to verify the performance of the anti-human secondary antibody. Additional PEG-AVP0458 control wells were also incubated with PEG-B-47 rabbit monoclonal antibody prior to addition of a Goat anti-rabbit IgG (H+L) horseradish peroxidase conjugate to verify the procedure for coating of the wells with PEG-AVP0458.

### Whole body clearance

Whole body clearance of ^124^I-PEG-AVP0458 (biological half-life T_1/2,biol_, and effective half-life T_1/2,eff_) was calculated from the whole body PET volumetric images of five patients obtained at the multiple imaging time points post infusion. Patient 2 was excluded from the analysis due to the extravasation of the injected dose.

The whole-body ROIs were delineated to encompass the whole-body regions in the images acquired at each imaging time points. The uptakes in the whole-body ROIs were normalized to the first imaging time point on Day 0. A time versus activity curve (TAC) was firstly generated from the retained radioactivity in the whole-body ROIs at each imaging time point. From this TAC, a mono-exponential clearance expression was fitted to obtain effective half-life, T_1/2,eff_, by the following equation:


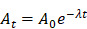


where *A_t_* is activity at time *t*, *A_0_* is activity at the first imaging time point, *λ* is the decay constant for the function.

### Radiation dosimetry

The radiation dosimetry method used is an adaptation of that promulgated by the MIRD (Medical Internal Radionuclide Dosimetry) Committee, accounting for the physical properties of the administered radionuclides (^124^I) as well as the biological properties (PK and biodistribution) of the radiopharmaceutical in individual patients 34. Serial whole-body PET and CT scans enabled derivation of tumour and normal-organ absorbed dose (mGy and mGy/mBq) estimates using ROI-derived time-activity data. Using the patient's total-body mass (in kg) and the 73.7-kg Standard Man organ masses, the total-body and organ ROI data [mean standard uptake values (SUVs)] were converted to activities (fraction of the injected dose). These image-derived time-activity data were fitted to exponential functions using a least-squares fitting algorithm and the resulting time-activity functions analytically integrated, incorporating the effect of physical decay of ^124^I to yield the time-integrated activity coefficients in MBq-hr/MBq in the organs and whole body. Time-integrated activity coefficients were used to calculate ^124^I-labeled PEG-AVP0458 mean absorbed doses to the organs (mGy/MBq) and effective dose (mSv/MBq) individually in the evaluable patients using a 73.7 kg adult male phantom in OLINDA 1.0 EXM program 35. Tumor self-dose was estimated as the mean absorbed dose in tumor using the sphere model in OLINDA EXM 1.0 Program.

### Statistical analyses

Biodistribution, tumor and normal organ dosimetry, whole body clearance and pharmacokinetic parameters were examined quantitatively and descriptive statistics such as mean, SD, and independent sample *t* tests were used to analyze these data. A *P* value of less than 0.05 was considered statistically significant.

## Results

### Production and characterization of PEG-AVP0458

**Figure 1:**
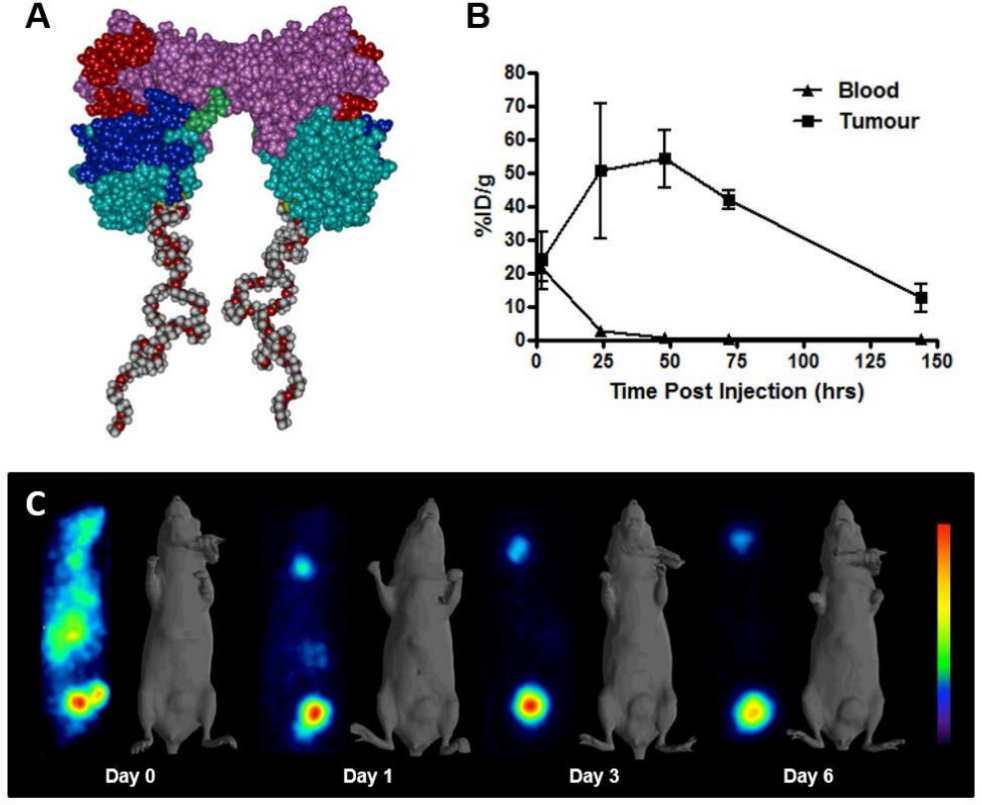
** Schema of PEG-AVP0458, and preclinical ^124^I- PEG-AVP0458 data.** (**A**) diagrammatic representation of PEG-AVP0458, showing heavy chain (purple) with CDR residues (red), light chain (aqua) and CDR residues (dark blue), linking residues (light green), PEG (grey and red chains). (**B**) The biodistribution of ^124^I-PEG-AVP0458, anti-TAG72 diabody over 6 days in LS174T xenografts and serum from BALB/c nude mice. Values are mean percent injected dose per gram (%ID/g) from groups of five mice; *bars,* SD. (**C**) Biodistribution Study. Representative images on Day 0 (4 hrs p.i.) Day 1, 3 and 6 of ^124^I- PEG-AVP0458 biodistribution in BALB/c nude mice bearing LS174T colon carcinoma xenografts in the left flank *(right side of each image*). Image set from each day comprise coronal whole body PET *(left panel)* and surface-rendered CT *(right panel).* High specific tumor uptake of ^124^I-PEG-AVP0458 is evident from Day 1 with normal tissue localisation corresponding to non-specific blood pool activity on Day 0 and anticipated thyroid uptake of ^124^I evident.

AVP0458 is a diabody, a non-covalent dimer of two single-chain variable fragments (scFv) with two antigen-binding Fv domains and two surface disulphides and is identical to the AVP04-50 used in previous preclinical studies 31 except that AVP0458 lacks the C-terminal His6-tail (Figure 1A). Following cGMP production, the binding activity and specificity of the pegylated diabody for TAG-72 was verified, initially using column shift assays to show that full binding activity was retained to a TAG-72 mimetic antigen (BSM-mucin) (Figure S1). *In vitro* biosensor analysis utilizing surface plasmon resonance (SPR) demonstrated that CC49, AVP0458 and PEG-AVP0458 competed for the same BSM epitope. BIAcore analyses of AVP0458 binding to BSM (sialyl-Tn antigen mimetic) determined an equilibrium dissociation affinity constant (K_D_) for AVP0458 of 9.23 nM (Figure S2). Immunohistochemical analysis of prostate cancer tissue showed comparable specificity and staining pattern for AVP0458 diabody and CC49 antibody binding to secretory product and cell cytoplasm in a series of prostate adenocarcinoma samples (Figure S3).

A fourteen-day toxicity study of intravenous single-dose PEG-AVP0458 (at doses of 0.0, 2.0, 6.7 and 20.0 mg/kg) was undertaken in Sprague-Dawley rats to assess the potential effect on vital organ functions including cardiovascular, respiratory and central nervous system. The control and high dose groups included ten males and ten females. The test product PEG-AVP0458 was well tolerated in this study, body weight and feed consumption were not affected and there were no adverse events, unexpected deaths, morbidity nor clinical signs of toxicity, including the highest dose which was up to fifty-fold higher than planned for the human study. A No Adverse Effect Level (NOAEL) dose of 20mg/kg was assigned for this study.

### ^124^I-PEG-AVP0458 preclinical model studies

^124^I-PEG-AVP-0458 was prepared in a stable form with retention of immunoreactivity and demonstrated >95% radiochemical purity. The biodistribution properties and PET imaging characteristics of ^124^I-PEG-AVP0458 were then evaluated in BALB/c nude mice bearing TAG-72 expressing LS174T colorectal cancer xenografts. High tumor uptake was observed, with prolonged tumor retention, and excellent imaging properties (Figure 1B-C). Minimal normal tissue uptake (only anticipated free ^124^I to unblocked thyroid) was observed. The mean uptake of ^124^I-PEG-AVP0458 by TAG-72 expressing LS-174T tumors reached a maximum of 54.12 ± 8.49 %ID/g by 48 hours post injection, with a tumor:blood ratio at 24 hrs of 21.2:1, at 48 hrs 60.8:1, and at 72 hrs 160.6:1 (Figure 1C) and serum half-life of T½ α = 6.77 hours, and T½ β = 29.72 hours.

### Clinical trial design

**Figure 2:**
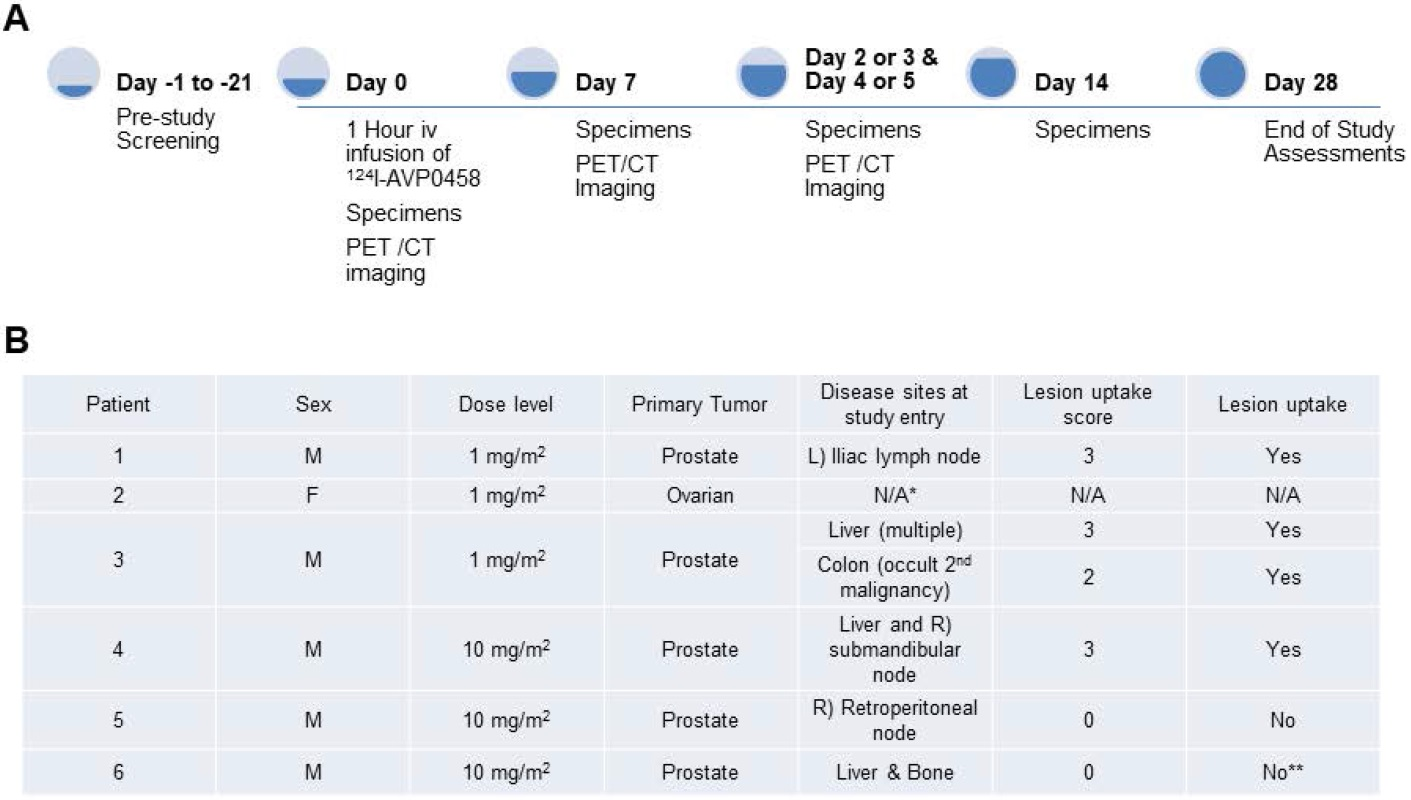
** Overview of first-in-human study. (A)** Clinical study events pre- and post- ^124^I-PEG-AVP0458 infusion denoting timing of acquisition of PET-CT images and collection of blood specimens. **(B)** Patient clinical demographics, and tumor lesion uptake and score. * Dose extravasated, not assessable (N/A); **Biopsy of liver lesion was TAG-72 -ve; true negative result.

A total of 6 patients were entered into this first-in-human trial, at the 1.0 mg/m^2^ (n=3) and 10 mg/m^2^ (n=3) dose levels. The choice of the two doses of PEG-AVP0458, 1 mg/m^2^ and 10 mg/m^2^, was based on our mouse model biodistribution studies, and prior trials of CC49 that showed that optimal tumor uptake and biodistribution occurred at 10 mg/m^2^ dose levels 24-26. We also established 1.0 mg/m^2^ as a suitable dose for optimal specific activity of ^124^I-AVP0458. The study schema is illustrated in Figure 2A. The demographics and clinical history of the patient population are summarised in Figure 2B.

### Patient safety evaluation

^124^I-PEG-AVP0458 was well tolerated for both 1 mg/m^2^ and 10 mg/m^2^ doses. The 1mg/m^2^ dose level patients were infused with 1.97 ± 0.31 mg (mean ± SD) AVP0458, and 10mg/m^2^ dose level were infused with 18.3 ± 1.76 mg AVP0458. No serious adverse events were reported. No adverse events occurred that were considered possibly related to ^124^I-PEG-AVP0458. Three patients reported adverse events, all Grade I or II, and which resolved with treatment (Table S1).

### Diabody biodistribution and dosimetry

**Figure 3:**
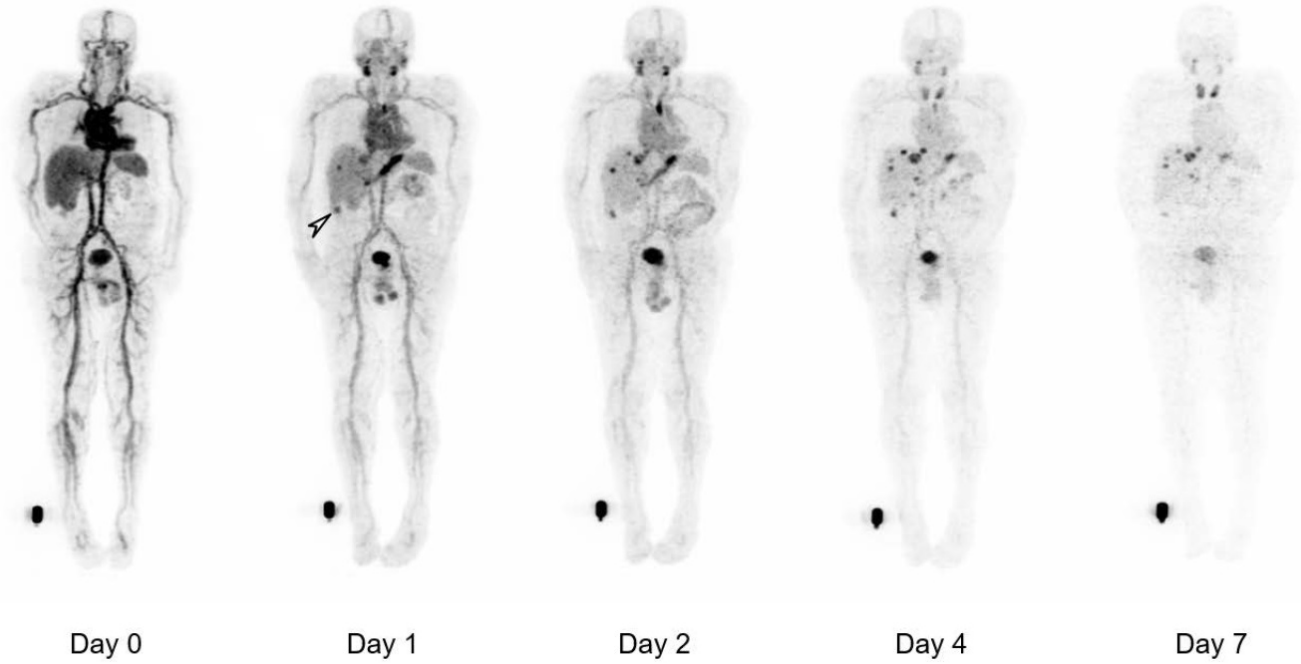
** Whole body biodistribution of ^124^I-PEG-AVP0458 in Patient 3 (1 mg/m^2^ dose level).** Sequential whole-body PET images acquired over one week following a single ^124^I-PEG-AVP0458 infusion showing gradual blood-pool clearance and no specific normal tissue uptake. Localisation of the diabody to sites of metastatic prostate cancer are evident from day 1 and an incidental TAG-72 positive colon tumor was also identified (*arrow*). Late visualization of the thyroid is likely to reflect free ^124^I due to late de-iodination of the agent.

**Figure 4:**
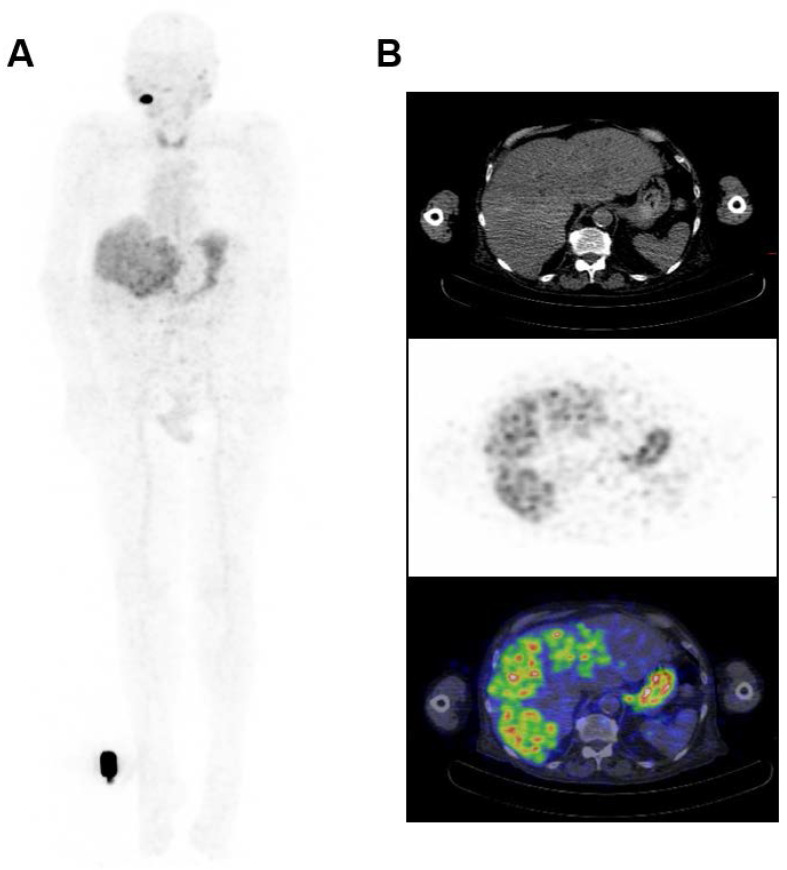
** Whole Body Biodistribution of ^124^I-PEG-AVP0458 (10mg/m^2^ dose level). (A)** Anterior Whole Body PET image on Day 5 in Patient 4. Transaxial images in **(B)** liver *(Upper panel - CT, Middle panel - PET, Lower panel -merged PET/CT)* showing excellent uptake of ^124^I-PEG-AVP0458 in extensive liver metastases of prostate cancer. Whole body images include reference standard by right foot in field of view.

The biodistribution of ^124^I-PEG-AVP0458 was assessable in 5 of 6 patients, as patient 2 (1 mg/m^2^ cohort) dose of ^124^I-PEG-AVP0458 was extravasated and rendering the patient data not evaluable for biodistribution or dosimetry analysis. Figure 2 includes qualitative tumor uptake results for individual patients. Whole body images and representative biodistribution pattern are presented in Figure 3 (1 mg/m^2^ dose level) and selected transaxial PET/CT sections to highlight metastatic prostate cancer tumor regions are presented in Figure 4 (10 mg/m^2^ dose level).

^124^I-PEG-AVP0458 PET imaging showed stable and consistent biodistribution across all patients at both dose levels, with gradual clearance from blood with time, and no significant normal tissue uptake. In particular, no discernible kidney uptake of ^124^I-PEG-AVP0458 was observed. Tumor uptake was rapid and evident by 1‑2 days post-injection, with sites of metastatic disease in lymph nodes and liver identified. Image-derived time-activity data were used to calculate the median time-integrated activity coefficients (residence time) of ^124^I-PEG-AVP0458 in all organs and tissues (Table S2). The organ receiving the highest mean absorbed dose was the thyroid gland (2.21 ± 0.61 mGy/MBq). Maximal tumor uptake observed was 12.2 × 10^-3^ %ID/mL at 7 days. The specific tumor absorbed dose ranged from 1.25 mGy/MBq to 4.75 mGy/MBq (mean ± SD = 2.87 ± 1.49 mGy/MBq), and the measured absorbed tumor dose ranged from 235.88 mGy to 833.06 mGy (mean ± SD = 605.32 + 322.83 mGy).

**Figure 5:**
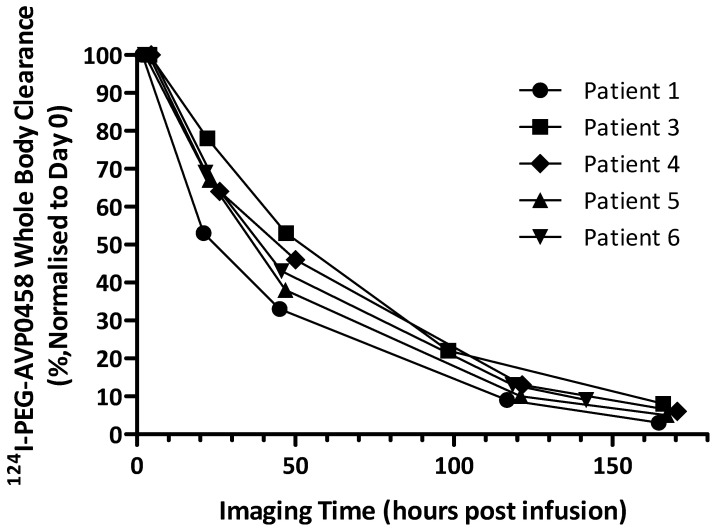
** Whole body clearance of ^124^I-PEG-AVP0458 in all patients.** Whole body clearance was calculated from acquired PET image data from Day 0, Day 1, Day 2 or 3, Day 4 or 5, and Day 6 or 7 post-^124^I-PEG-AVP0458 infusion imaging time points.

Whole body clearance of ^124^I-PEG-AVP0458 was consistent among patients, and across dose levels (Figure 5). There were no statistically significant differences between the two dose levels. The mean effective half-life of ^124^I-PEG-AVP0458 (measuring the time for decrease of ^124^I-PEG-AVP0458 to half the initial value by both biological clearance and radioactive decay) was 56.74 ± 8.80 hours in the 1 mg/m^2^ dose group, and 57.84 ± 2.31 hours in the 10 mg/m^2^ dose group (*P*=0.97). The mean biological half-life of ^124^I-PEG-AVP0458 (measuring only the biological clearance of ^124^I-PEG-AVP0458) was 136.61 ± 21.62 hr in the 1 mg/m^2^ dose group, and 137.24 ± 12.89 hr in the 10 mg/m^2^ dose group (*P* =0.89). The effective whole body dose was calculated at 0.56 ± 0.09 mSv/MBq.

### Immunogenicity

All the samples analyzed from all the patients were negative for anti-diabody antibodies, indicating lack of immune response in the patients to the PEG-AVP0458.

### Pharmacokinetics

Serum concentration values of ^124^I-PEG-AVP0458 showed consistent values between patients, and there was no significant difference in T½α and T½β between dose levels (Table 1). AUC and C_max_ values were proportionally increased between dose levels. The overall mean (± SD) PK parameters for ^124^I-PEG-AVP0458 were T½α = 5.10 ± 4.58 hours, T½β = 46.19 ± 13.06 hours, CL = 137.10 ± 47.25 mL*/*hr and V1 = 4.67 ± 1.25 L (Table 1).

The AUC and C_max_ results from the ELISA analyses of protein PEG-AVP0458 and radioactivity measurements of ^124^I-PEG-AVP0458 in the patient's serum samples were in good agreement at both dose levels (Table S3). There were no statistically significant differences between these parameters for the study measurements.

## Discussion

We have shown that a pegylated diabody can be engineered with characteristics suitable for successful translation into clinical studies. After encouraging pre-clinical testing showing in-vivo stability and tumor targeting capability of ^124^I-PEG-AVP0458, we conducted a first-in-human biodistribution study which demonstrated the cancer targeting capabilities of pegylated diabodies, and the ability to achieve rapid, high targeting of tumor without significant normal tissue or kidney retention. The selective targeting demonstrated, as well as the ability to image agent distribution to tumors throughout the body, supports their potential capability for use for payload delivery, and for theranostic use in patients.

Unmodified diabodies have been extensively evaluated in biodistribution, tumor uptake and pharmacokinetic studies, using many different xenograft models. In general, stable bivalent diabodies have proved to be excellent imaging tools, with reasonable tumor uptake combined with rapid systemic clearance, achieving high tumor:blood ratios after 4 hours, which translates to ideal PET/SPECT imaging pharmacokinetics 7,15. However, their rapid systemic clearance through kidney filtration has a significant impact in reducing both total tumor uptake and longevity in tumors (area-under-curve), compared to intact antibodies. Effective targeted tumour therapy requires both efficient delivery and retention of cytotoxic payloads, whether these are radioisotopes or chelated radionuclides or cytotoxic (ADC) drugs 1-6. We have previously demonstrated in animal models that pegylation of diabodies was an effective means of avoiding this unwanted first-pass renal clearance, whilst retaining both the excellent tumor penetration properties of diabodies and providing an ideal, intermediate, systemic clearance rate 30,31. Pegylated diabodies have also been shown to deliver drugs to tumors in preclinical models with marked inhibition of tumor growth 24,36. These properties of pegylated diabodies, compared to intact antibodies, make them potentially ideal delivery vehicles for tumor-targeted radionuclide or cytotoxic payloads with a hope of providing a reduction in the off-target toxicity observed with intact conjugated antibodies.

In our preclinical studies using TAG-72 positive human cancer xenografted mice, clinical grade ^124^I-PEG-AVP0458 was stably generated with retention of immunoreactivity *in vitro* and achieved high tumor uptake *in vivo* and serum half-life of T½α=6.77 hours, and T½β=29.72 hours. These properties, together with minimal binding to non-tumor tissue, particularly liver and kidney, resulted in very high tumor:blood ratios at 24-48 hours post-administration and supported the use of PEG-AVP0458 as a specific targeting strategy for TAG-72 expressing tumors and, when radiolabeled with ^124^I, an ideal radioimaging reagent for PET. We selected ^124^I based on the known low internalisation rate of TAG-72 and prior animal model and clinical studies using radioiodinated forms of CC49 24-25. While ^124^I-labeled antibodies may demonstrate less retention within tumors compared to radiometals due to dehalogenation once internalized, the data does confirm the excellent tumor targeting properties of PEG-AVP0458, which could be exploited for payload/drug delivery.

The high tumor uptake and tumor:blood ratios observed in our xenograft studies with pegylated diabodies compares favourably with the results of other engineered antibody formats, including naked ~55kDa diabodies 7, ~85 kDa minibodies 37 and various scFv fusions and extensions to increase the systemic half-life such as scFv-Fc and scFv-albumin 15,38-39. Compared to these other engineered antibody formats, albeit against different tumor antigens and xenograft models, our pegylated diabody PEG-AVP0458 exhibited the highest tumor uptake ratios observed (over 50% ID/g at 24 and 48 hours), high tumor:blood ratios, and with prolonged retention in tumor. This preclinical data provided justification to extend into human studies.

In this first-in-human trial, PEG-AVP0458 was shown to be safe and well tolerated in prostate and ovarian cancer patients, with no adverse events related to study drug observed following the single administration of PEG-AVP0458 labeled with the PET isotope ^124^I. A pivotal observation from our clinical trial was the observation that PEG-AVP0458 did not significantly localize to any normal tissue, and in particular no kidney uptake was observed. Some evidence of ^124^I-PEG-AVP0458 catabolism through liver clearance was noted. Thyroid uptake was non-specific and due to free ^124^I accumulation, which is often observed following radioiodine-antibody administration, despite blocking with oral Lugol's iodine 24,25. The high uptake of free ^124^I in thyroid tissue would be an impediment to using radiohalides linked to PEG-AVP0458 for therapeutic indications. This biodistribution data is in contrast to other clinical trials of small molecular weight constructs, including many peptides (below 10kDa), an scFv (~27kDa) and an scFv dimer (~55kDa) which all show kidney uptake and rapid blood clearance. Specifically, in these human studies, peptides (below 10kDa) clear systemically with half-lives of minutes 40, whilst an scFv (~27kDa) had a half-life of 30 minutes 41 and the scFv dimer blinatumomab (an ~55kDa BiTE^TM^) had a half-life of 1.25 hours 42. The TNF-targeted Fab (~ 50kDa) certolizumab exhibited fast systemic clearance and was only therapeutically effective as a pegylated derivative with longer half-life 43. Of the larger engineered formats, a ~90kDa F(ab')_2_ and an ~80kDa scFv-CH3 dimer (minibody) both avoided renal clearance and exhibited systemic half-lives of ~20hr and ~29hr respectively 44. Taken together, there is a clinical imperative to design and evaluate small antibody fragments that avoid first-pass kidney clearance (particularly for payloads such as residualizing radiometals) whilst retaining high tumor penetration in order to provide effective delivery of therapeutic payloads.

Both biodistribution and dosimetry analysis in our first-in-human trial confirmed no specific normal tissue uptake of ^124^I-PEG-AVP0458, and no saturable normal tissue compartment, at both the 1 mg/m^2^ and 10 mg/m^2^ dose levels. High tumor uptake of metastatic prostate cancer was observed in liver metastases and tumor involved lymph nodes, was rapid, and an incidental TAG-72 colon tumor was also identified by biodistribution analysis of PET images. The mean effective dose of ^124^I-PEG-AVP0458 was in good agreement with published data of other ^124^I-labeled tracers. Furthermore, through dosimetry analyses, ^124^I-PEG-AVP0458 demonstrated selective targeting of tumor, which has quantitative uptake comparable or superior to intact humanised antibodies but with a lower serum AUC. This superior tumor:serum AUC indicates that ^124^I-PEG-AVP0458 is ideally suited to theranostic and payload delivery strategies in patients with TAG-72 positive tumors. In view of the ability to target drugs specifically to tumor in mouse models with potent therapeutic effect with AVP0458-drug-conjugates 36, the confirmation of tumor targeting in this first-in-human trial demonstrates the validity of this approach for future trials where molecular imaging of AVP0458 uptake in tumor could assist with patient selection. The data also supports the potential development of therapeutic radioisotope labeled PEG-AVP0458 with alpha or beta particle emitters for cancer therapy.

No significant differences between dose cohorts were observed in ^124^I-PEG-AVP0458 T½α and T½β clearance, or volume of central compartment. The ^124^I-PEG-AVP0458 terminal (β) half-life of 46.19 ± 13.06 hours was highly comparable to the reported elimination half-life of 44.9 ± 10.6 hours for ^131^I-murine CC49 25 and markedly longer than the 10.5 hours for CC49 scFv 41. In comparison, the radiolabelled bivalent minibody construct chimeric T84.66 targeting the carcinoembryonic antigen (cT84.66, 80 kDa) demonstrated a mean blood residence time of 29.8 hours 44*.* The whole-body clearance (biological T_1/2_, and effective T_1/2_) showed no difference between dose levels, and the observed Teff of 57.40 ± 4.73 hour for ^124^I-PEG-AVP0458 compared favourably with prior observations for Yttrium-90 labelled HuCC49DCh2, a CH2 domain deleted and humanized CC49 where the median (range) effective clearance half-life was 51.8 hours (47.6-55.8) for the whole body 45.

There are some limitations to our results, as we did not include a negative control tumor for our preclinical biodistribution experiments, although the degree of uptake in tumor would support the specific uptake related to TAG-72 expression in tumor. In addition, only five prostate cancer patients were evaluable for the biodistribution and pharmacokinetic analysis in the first-in-human trial. This does impact on the ability to confirm any differences in radiation dosimetry based on gender. Nevertheless the reproducibility of the biodistribution data is highly promising, and consistency of pharmacokinetic results indicate PEG-AVP0458 is stable in-vivo and has high tumor:blood ratios in both animal models and patients.

Taken together, the novel, site-directed surface-pegylation of the AVP0458 diabody and, now, the clear demonstration of high tumor uptake in cancer patients, mid-range systemic clearance and no specific off-target binding, has shown that pegylated diabodies are potentially an ideal platform for delivery of radionuclide or cytotoxic payloads, and as theranostic probes for imaging. This approach could be extended to a broad range of tumor cell and microenvironment targets, where antibody modification into a pegylated diabody format will allow high tumor to normal tissue ratios and potentially improve therapeutic outcomes.

## Supplementary Material

Supplementary tables and figures.

## Figures and Tables

**Table 1:** ^124^I-PEG-AVP0458 pharmacokinetic parameters (Mean ± SD) in all patients, and statistical comparison between dose levels

Parameter (units)	T½ α (hr)	T½ β (hr)	Cmax (µg/mL)	AUC (hr*ug/mL)	V1 (L)	CL (mL/hr)
All Subjects (n=5)	5.1 ± 4.58	46.19 ± 13.06	2.77 ± 2.2	97.19 ± 77.91	4.67 ± 1.25	137.1 ± 47.25
Dose Cohort, 1 mg/m^2^ (n=2)	4.88 ± 2.57	43.03 ± 1.42	0.43 ± 0.09	14.56 ± 5.23	5.17 ± 1.82	157.97 ±76.23
Dose Cohort, 10 mg/m^2^ (n=3)	5.26 ±6.2	48.3 ± 17.98	4.33 ±0.71	152.27 ±27.36	4.33 ±1.01	123.2 ±28.87
T-Test comparing, 2 dose levels	*P* = 0.942	*P* = 0.721	*P* = 0.005*	*P* = 0.007*	*P* = 0.541	*P* = 0.501

## Abbreviations

ADC: antibody-drug conjugate
AUC: area under curve
CT: computed tomography
ELISA: enzyme-linked immunosorbant assay
HRP: horseradish peroxidase
IHC: immunohistochemistry
IRB: Institutional Review Board
kDa: kilodalton
PEG: pegylated
PET: positron emission tomography
scFv: single chain Fv fragment
TAG-72: tumor-associated glycoprotein 72
