# Supplementary material for: First clinical study of a pegylated diabody 124I-labeled PEG-AVP0458 in patients with tumor-associated glycoprotein 72 positive cancers
Source: Theranostics. 2020 Sep 15;10(25):11404–15. doi: 10.7150/thno.49422 (PMC7545991; doi:10.7150/thno.49422)
Supplement: Supplementary file 1 — Supplementary tables and figures. [file thnov10p11404s1.pdf]

Table S1. Adverse Events

**Patient**	**Adverse Event**	**Severity**	**Relationship to Study Drug**
1	pyrexia	Grade II	unrelated
1	vomiting	Grade I	unrelated
1	neutrophilia	Grade I	unrelated
2	extravasation blood	Grade I	unrelated
2	contusion	Grade I	unrelated
4	respiratory tract infection	Grade II	unrelated

Table S2. Normal organ dosimetry analysis performed using OLINDA 1.0 EXM

Organ / Parameter	Absorbed Dose (mGy/MBq)					
	S00015	S00026	S00028	S00030	S00031	Mean ± SD (n = 5)
**Adrenal Glands**	0.30	0.39	0.56	0.26	0.45	0.39 ± 0.12
**Brain**	0.16	0.19	0.22	0.12	0.21	0.18 ± 0.04
**Breasts**	0.16	0.22	0.32	0.16	0.27	0.23 ± 0.07
**Gallbladder Wall**	0.49	0.61	0.85	0.40	0.48	0.56 ± 0.18
**Lower Large Intestine Wall**	0.54	0.57	0.75	0.45	0.63	0.59 ± 0.11
**Small Intestine**	0.59	0.62	0.68	0.32	0.64	0.57 ± 0.14
**Stomach Wall**	0.58	0.68	1.05	0.56	0.64	0.70 ± 0.20
**Upper Large Intestine Wall**	0.50	0.69	0.75	0.38	0.62	0.59 ± 0.15
**Heart Wall**	0.67	0.84	0.71	0.59	0.88	0.74 ± 0.12
**Kidneys**	0.82	0.71	1.00	0.47	0.79	0.76 ± 0.19
**Liver**	0.54	0.79	1.51	0.40	0.67	0.78 ± 0.43
**Lungs**	0.29	0.39	0.42	0.27	0.37	0.35 ± 0.06
**Muscle**	0.23	0.35	0.38	0.23	0.39	0.32 ± 0.08
**Ovaries**	0.32	0.41	0.50	0.27	0.47	0.39 ± 0.10
**Pancreas**	0.77	0.59	0.79	0.51	0.82	0.70 ± 0.14
**Red Marrow**	0.37	0.39	0.51	0.26	0.55	0.42 ± 0.12
**Osteogenic Cells**	0.36	0.43	0.58	0.30	0.57	0.45 ± 0.13
**Skin**	0.14	0.20	0.27	0.14	0.24	0.20 ± 0.06
**Spleen**	0.85	0.75	0.81	0.50	0.84	0.75 ± 0.15
**Testes**	0.19	0.27	0.35	0.19	0.33	0.27 ± 0.08
**Thymus**	0.21	0.30	0.39	0.21	0.36	0.30 ± 0.08
**Thyroid**	2.56	1.39	1.76	2.82	2.54	2.21 ± 0.61
**Urinary Bladder Wall**	0.81	1.12	0.99	0.58	1.07	0.91 ± 0.22
**Uterus**	0.32	0.43	0.51	0.27	0.48	0.40 ± 0.10
**Total Body**	0.26	0.36	0.45	0.24	0.41	0.34 ± 0.09
**ED Equivalent (mSv/MBq)**	0.51	0.54	0.68	0.41	0.63	0.55 ± 0.10
**ED (mSv/MBq)**	0.51	0.53	0.69	0.46	0.62	0.56 ± 0.09

.

The organs receiving the highest mean absorbed dose were the thyroid gland (2.21 ± 0.61 mGy/MBq), the urinary bladder wall (0.91 ± 0.22 mGy/MBq), the liver (0.78 ± 0.43 mGy/MBq), and the spleen (0.75 ± 0.15 mGy/MBq). The lowest mean absorbed dose was observed in the brain (0.18 ± 0.04 mGy/MBq). The results are consistent with no specific uptake of ^{124}I-PEG-AVP0458 in normal organs, and some free ^{124}I uptake in thyroid.

Table S3. Pharmacokinetic parameters AUC and Cmax for all ^{124}I-PEG-AVP0458 patients compared with ELISA PEG-AVP0458 data for all patients.

**Cohort mg/m^2**	**Patient**	**AUC (hr*μg/mL) ELISA**	**AUC (hr*μg/mL) ^{124}I**	**Cmax (μg/mL) ELISA**	**Cmax (μg/mL) ^{124}I**
**1**	1	2.9	10.86	0.1	0.36
	3	6.9	18.26	0.3	0.49
	**Mean**	**4.9**	**14.56**	**0.2**	**0.43**
	**SD**	**2.8**	**5.23**	**0.1**	**0.09**
T Test, P Value		P = 0.148		P = 0.137	
**10**	4	162.8	151.65	3.2	5.14
	5	137.5	125.23	2.8	4.08
	6	116.4	179.94	3.3	3.78
	**Mean**	**138.9**	**152.3**	**3.1**	**4.3**
	**SD**	**23.2**	**27.4**	**0.3**	**0.7**
T Test, P Value		P = 0.429		P = 0.052	

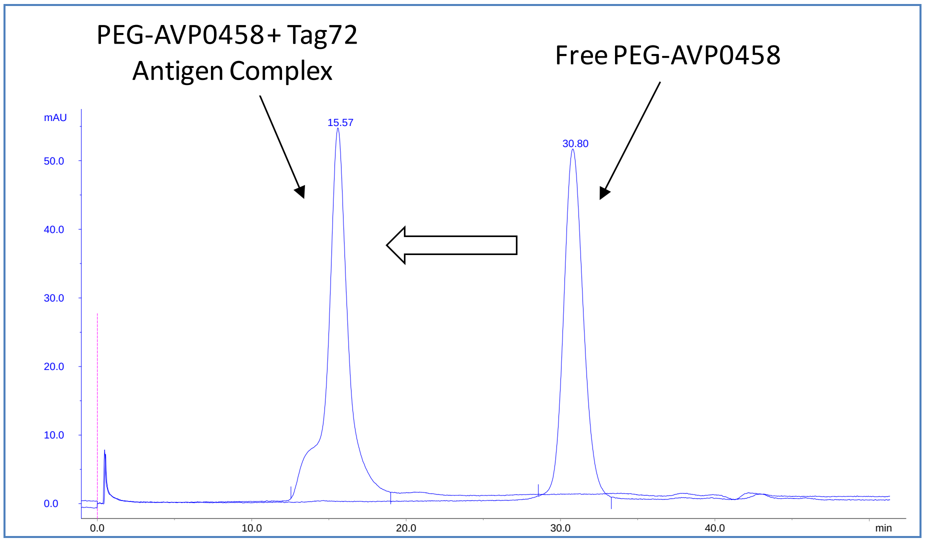

Figure S1. Column Shift assays to BSM (bovine submaxillary mucin), demonstrating full binding activity of AVP0458 retained after PEGylation.

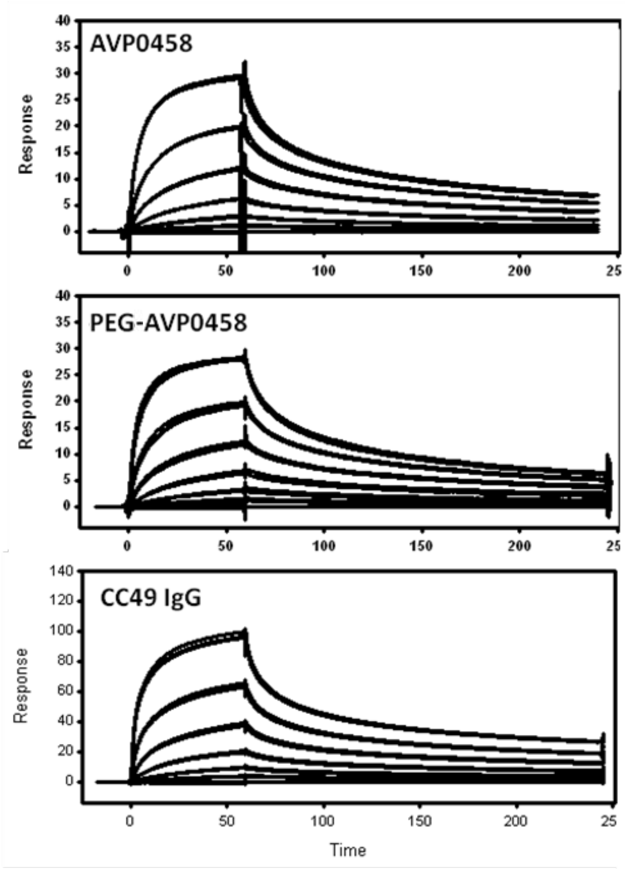

Figure S2. Biosensor curves for AVP0456, PEG-AVP0458, and CC49 against TAG-72 antigen.

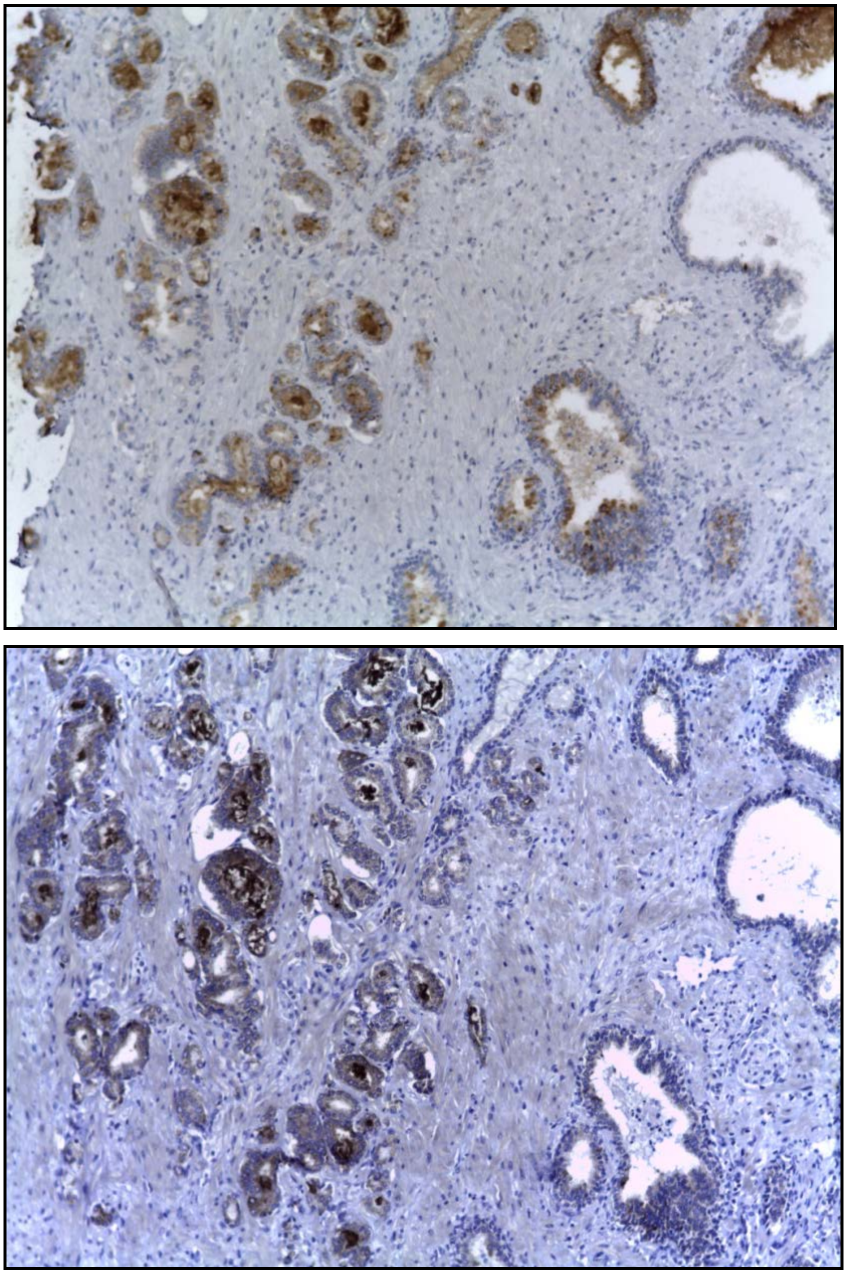

Figure S3. Immunohistochemical staining of serial sections of FFPE prostate cancer tissue with anti-TAG72 CC49 (top panel) and AVP0458-biotin (bottom panel). Original magnification, 200×.
